# Risk factors for future repeat abdominal surgery

**DOI:** 10.1007/s00423-016-1414-3

**Published:** 2016-04-13

**Authors:** Chema Strik, Martijn W. J. Stommel, Laura J. Schipper, Harry van Goor, Richard P. G. ten Broek

**Affiliations:** Department of Surgery, Radboud University Medical Center, P.O. Box 9101, 6500 HB Nijmegen, The Netherlands

**Keywords:** Abdominal adhesions, Abdominal surgery, Postoperative complications, Risk factors

## Abstract

**Purpose:**

Today, 40 to 66 % of elective procedures in abdominal surgery are reoperations. Reoperations show increased operative time and risk for intraoperative and postoperative complications, mainly due to the need to perform adhesiolysis. It is important to understand which patients will require repeat surgery for optimal utilization and implementation of anti-adhesive strategies. Our aim is to assess the incidence and identify risk factors for repeat abdominal surgery.

**Methods:**

This is the long-term follow-up of a prospective cohort study (Laparotomy or Laparoscopy and Adhesions (LAPAD) study; clinicaltrials.gov NCT01236625). Patients undergoing elective abdominal surgery were included. Primary outcome was future repeat abdominal surgery and was defined as any operation where the peritoneal cavity is reopened. Multivariable logistic regression analysis was used to identify risk factors.

**Results:**

Six hundred four (88 %) out of 715 patients were included; median duration of follow-up was 46 months. One hundred sixty (27 %) patients required repeat abdominal surgery and underwent a total of 234 operations. The indication for repeat surgery was malignant disease recurrence in 49 (21 %), incisional hernia in 41 (18 %), and indications unrelated to the index surgery in 58 (25 %) operations. Older age (OR 0.98; *p* 0.002) and esophageal malignancy (OR 0.21; *p* 0.034) significantly reduced the risk of undergoing repeat abdominal surgery. Female sex (OR 1.53; *p* 0.046) and hepatic malignancy as indication for surgery (OR 2.08; *p* 0.049) significantly increased the risk of requiring repeat abdominal surgery.

**Conclusions:**

One in four patients will require repeat surgery within 4 years after elective abdominal surgery. Lower age, female sex, and hepatic malignancy are significant risk factors for requiring repeat abdominal surgery.

## Introduction

An increasing number of patients undergo abdominal surgery multiple times during their lifetime, due to a higher life expectancy and advances in surgical technology; this is expected to increase even further [1, 2]. Today, as many as 40 to 66 % of elective procedures in general surgery are reoperations [3–5]. It is estimated that 10 to 37 % of patients undergoing elective abdominal surgery will require repeat abdominal surgery and might thus benefit from the use of anti-adhesive barriers [6, 7]. During reoperations, the need for adhesiolysis results in increased operative time, a 6 to 10 % incidence of inadvertent bowel injury, and a longer and more complicated convalescence. The risk for bowel injuries is amplified by each consecutive laparotomy and can be as high as 50 % [8]. Furthermore, increased postoperative mortality and higher health care costs are reported especially when adhesiolysis resulted in bowel injury [5, 9].

It is important to understand which patients will require repeat surgery for the optimal resource utilization and implementation of anti-adhesive strategies in order to reduce adhesiolysis-related complications. A recent systematic review and meta-analyses of four commercially available anti-adhesive barriers demonstrated that these barriers effectively reduce the incidence and severity of adhesions and operative time [10].

Currently, it is unknown which patients are at risk for undergoing repeat abdominal surgery. The risk of repeat abdominal surgery has only been investigated in a number of disease-specific cohorts which assessed risk factors for undergoing repeat surgery for disease recurrence [11, 12]. Population-based studies only focused on the incidence and did not provide patient-specific risk factors for undergoing repeat surgery [6, 7].

The aim of this study is to analyze patterns of repeat abdominal surgery during long-term follow-up and identify risk factors for requiring repeat abdominal surgery in a cohort of patients undergoing elective abdominal surgery.

## Material and methods

### Study design and patients

This is a follow-up study of the prospective “Laparotomy or Laparoscopy and Adhesions (LAPAD) study study,” clinicaltrials.gov registration number NCT01236625). Detailed methods of the LAPAD study are reported recently [5]. The LAPAD study included all patients admitted to the surgical ward of the Radboud University Medical Center for elective laparotomy or laparoscopy between June 2008 and June 2010. Demographics, preoperative surgical factors, and medical patient factors were prospectively collected. Patients who deceased within 30 days after discharge of the index admission were excluded from this study. Data on endpoints were gathered from 30 days after discharge until November 2013. For patients with multiple operations included in the LAPAD study, data was gathered from the last included operation. Patients and their general practitioners were contacted separately, and a questionnaire was sent regarding admissions to the departments of surgery for hospitalization for repeat abdominal surgery and episodes of bowel obstruction. Data was collected from medical records of hospitals and nursing homes when applicable. A waiver was obtained from the medical ethical committee of the Radboud University Medical Center (registration number 2013/097) for this study.

### Variables

Baseline demographics included sex, age, body mass index, American Society of Anesthesiologists classification, P-POSSUM score, presence of malignancy, number of previous laparotomies (0, 1 or 2, ≥3) and laparoscopies (0 and 1 or more), anatomical location of previous surgery and index operation (lower gastrointestinal, abdominal wall, other), and surgical approach (median, subcostal, other incision, and laparoscopy). Data on intraoperative factors collected were adhesiolysis time (0–30, ≥31 min), complete adhesiolysis defined as all peritoneal adhesions lysed, severity of adhesions underneath the incision, operative area and other abdominal areas according to the Zühlke classification [13] comprising 0, 1, and 2 as no or mild adhesions and 3 and 4 as severe adhesions, the location of adhesions (upper and lower abdomen) and any iatrogenic organ injury due to adhesiolysis, estimated blood loss, and the creation of an ostomy at the end of surgery. Postoperative data collected was the incidence of any intraabdominal complication within 30 days of the index operation, comprising intraabdominal sepsis, abscess, anastomotic leakage, fistula, delayed diagnosed perforation, hemorrhage, and a relaparotomy or relaparoscopy.

### Endpoints

Repeat abdominal surgery was defined as any operation where the peritoneal cavity is opened. In this study, we analyzed reoperations during the long-term follow-up (after 30 days from discharge). Immediate reoperations for serious adverse events of the index operation have previously been described [5]. Repeat abdominal surgeries were categorized in planned or unplanned operations. Planned repeat operations were defined as all repeat operations that are part of a staged treatment strategy (e.g., closure of a protective loop ileostomy or staged resection of synchronous hepatic metastasis from a colorectal carcinoma in situ). An operation was defined as unplanned if it was not part of the initial treatment strategy. The number of laparotomies and laparoscopies, the time interval between last included surgery and repeat abdominal surgery, surgical approach (open or laparoscopic), anatomical location, and indication for repeat surgery were registered. Indications for repeat abdominal surgery were categorized as malignant disease recurrence comprising both locoregional recurrence and distant metastasis, incisional or parastomal hernia, emergency laparotomy, adhesive small bowel obstruction or adhesiolysis for abdominal pain, ostomy closure \including loop ileostomy closure, relocation of ostomy, new ostomy creation for any reason, new malignancy, and other indications. For patients who required multiple operations, the date, surgical approach, and indication for surgery were registered separately.

### Statistical methods

Continuous variables are presented as means with standard deviation or medians with interquartile range if non-normal distribution. Dichotomous or categorical variables are presented as absolute numbers and percentages.

Univariable and multivariable logistic regression analysis was performed to identify risk factors for unplanned repeat abdominal surgery. All variables, with a *p* value of ≤0.10, were analyzed using a multivariable logistic regression analysis with stepwise backward selection, *p* entry ≤0.10 and *p* stay ≤0.10. The odds ratio, the 95 % confidence interval of the odds ratio, and the *p* value of risk factors are presented. The area under the receiver operating characteristic (ROC) curve (AUC) was used to quantify the predictive value of the logistic regression analysis.

A Kaplan-Meier analysis was performed showing the cumulative hazard risk of patients requiring repeat abdominal surgery over time.

A value of *p* ≤ 0.05 was considered significant. Statistical analysis was performed using SPSS for Windows version 20.0 software (SPSS, Chicago, IL). There was only minimal missing data; thus, we excluded per analysis those cases with missing data.

## Results

**Fig. 1:**
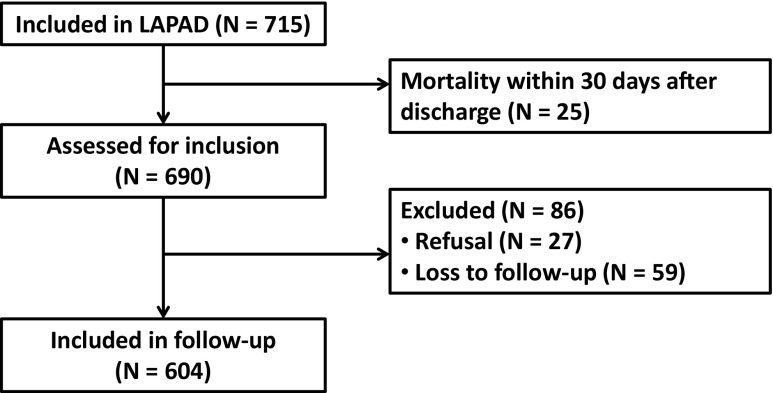
Flowchart

Seven hundred fifteen patients were eligible for inclusion in this study; 25 patients died within 30 days after discharge of the index admission terminating their follow-up. Out of 86 patients that were excluded, 27 patients declined to participate in follow-up and 59 patients were lost to follow-up, leaving 604 (88 %) patients for inclusion (Fig. 1). The median duration of follow-up was 46 (IQR 33–54) months.

### Baseline characteristics at index operation and incidence of repeat abdominal surgery

Table 1 shows the baseline characteristics. Mean (SD) age was 59 (±14), and 343 (57 %) patients were male. A median incision was used in 392 patients; a subcostal in 86 and other incisions were used in 70 patients, whereas a laparoscopic procedure was performed in 56 patients. Severe adhesions in the operative area were seen in 187 (31 %) of the patients. An ostomy was created in 107 (17 %) patients. Fifty-three (9 %) patients developed a postoperative intraabdominal complication.

**Table 1:** Baseline characteristics

Patient factors	
Sex	
Male	343 (57 %)
Female	261 (43 %)
Age*	59 ± 14
BMI*	25.7 ± 4.4
Smoking status	
Non-smoker	77 (34 %)
Ex-smoker	111 (50 %)
Smoker	36 (16 %)
Physiologic-POSSUM score^†^	16 (14–20)
ASA score	
1	104 (17 %)
2	370 (61 %)
3	130 (22 %)
Diagnosis of IBD	65 (11 %)
Number of previous laparotomies	
0	220 (36 %)
1 or 2	255 (42 %)
≥3	129 (21 %)
Previous laparoscopy	
Yes	531 (88 %)
No	73 (12 %)
Anatomical location previous surgery	
Upper GI	31 (5 %)
Lower GI	291 (48 %)
Abdominal wall	115 (19 %)
HPB	66 (11 %)
Other	200 (33 %)
Malignancy as indication for surgery	
Colorectal	108 (18 %)
Hepatic	68 (11 %)
Esophageal	42 (7 %)
Other	85 (14 %)
Benign indication for surgery	
Ventral hernia	104 (17 %)
Fistula	35 (6 %)
Other	194 (32 %)
Anatomical location index operation	
Lower GI	264 (44 %)
Abdominal wall	102 (17 %)
Other	238 (49 %)
Surgical approach	
Median	392 (65 %)
Subcostal	86 (14 %)
Other	70 (12 %)
Laparoscopy	56 (9 %)
Presence of adhesions	
Yes	379 (63 %)
No	225 (37 %)
Adhesiolysis time (minutes)	
0–30	474 (79 %)
>31	130 (21 %)
Adhesion severity underneath incision	
No or mild adhesions	419 (70 %)
Severe adhesions	178 (30 %)
Missing data	7 (1 %)
Adhesion severity operative area	
No or mild adhesions	407 (69 %)
Severe adhesions	187 (31 %)
Missing data	10 (2 %)
Adhesion severity other abdominal areas	
No or mild adhesions	460 (78 %)
Severe adhesions	127 (22 %)
Missing data	17 (3 %)
Iatrogenic organ injury due to adhesiolysis	148 (25 %)
Ostomy created	
Ileostomy	69 (11 %)
Colostomy	38 (6 %)
Wound classification	
Clean	237 (39 %)
Clean-contaminated	325 (54 %)
Contaminated	39 (7 %)
Dirty	3 (1 %)
Length of surgery	202 (145–269)
Intraabdominal complication	53 (9 %)
Relaparotomy	51 (9 %)

*mean values with a standard deviation

^†^a median value with an interquartile range of 25–75 %

Patients that were excluded were significantly younger (mean 55 years of age vs. 59 years; *p* 0.04), had more often a diagnosis of inflammatory bowel disease (17 (20 %) vs. 65 (11 %); *p* 0.01), and had less often an esophageal malignancy (4 (5 %) vs. 85 (14 %); *p* 0.02) in comparison to patients included in the follow-up study. Other baseline characteristics did not show significant differences (results not shown).

**Fig. 2:**
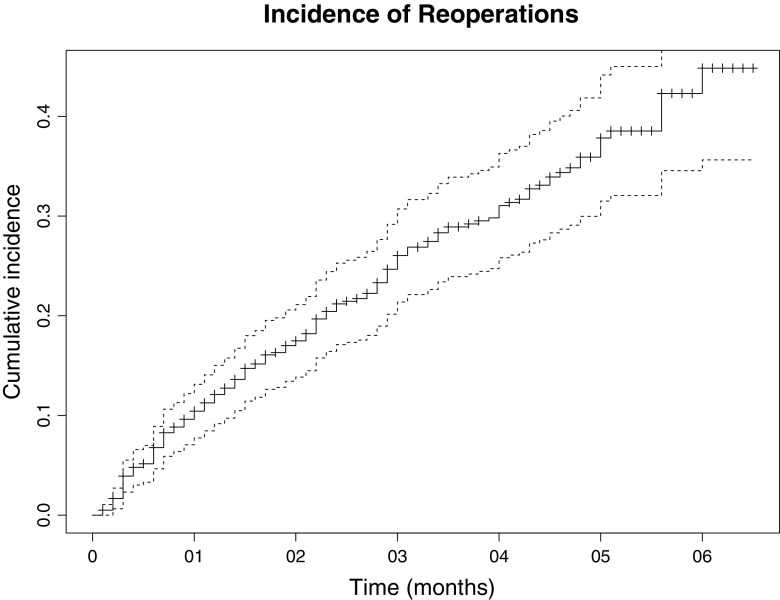
Cumulative risk over time for requiring repeat abdominal surgery; straight black line represents the mean cumulative incidence; the dashed line represents the 95 % confidence interval of the mean cumulative incidence

The incidence and characteristics of repeat abdominal surgery are shown in Table 2. One hundred sixty (27 %) patients underwent a total of 234 repeat abdominal operations, 108 (18 %) patients had one laparotomy, 29 (5 %) had two laparotomies, 16 (3 %) patients underwent three or more laparotomies, and 14 (2 %) patients required a laparoscopy. The cumulative incidence of repeat abdominal surgery after 2 years is 20 % (Fig. 2). One hundred ninety-six (84 %) operations were unplanned, and 38 operations were staged procedures of which 32 (84 %) were loop ileostomy closures. One hundred thirty-four (22 %) patients underwent at least one unplanned repeat abdominal operation. The anatomical location of repeat surgery was most often the lower gastrointestinal tract in 98 (45 %) and abdominal wall in 49 (23 %). The indication for repeat surgery was malignant disease recurrence in 49 (21 %), incisional or parastomal hernia in 41 (18 %), and other indications in 58 (25 %) operations. Other indications comprised predominately of open or laparoscopic cholecystectomy (28 % of other indications), results not shown. Three patients required a protective loop ileostomy during an unplanned repeat operation, which needed subsequent closure. Tables 3, 4, and 5 show the incidence of unplanned repeat abdominal surgery stratified for the anatomical location of the index operation, demonstrating that the abdominal wall (25 %) and lower gastrointestinal tract (24 %) have the highest incidence, although this did not reach a statistical significant difference (*p* = 0.53).

**Table 2:** The number of patients that underwent repeat abdominal surgery as well as the anatomical location and indication of repeat abdominal surgery

Repeat surgery	
Patients undergoing repeat surgery	
Yes	160 (27 %)
No	444 (73 %)
Patients undergoing unplanned repeat surgery	
Yes	134 (22 %)
No	470 (78 %)
Number of laparotomies	
1	108 (18 %)
2	29 (5 %)
≥3 (3–5)	16 (3 %)
Laparoscopy	
Yes	14 (2 %)
No	590 (98 %)
Number of planned operations	38 (16 %)
Closure protective loop ileostomy	32
Staged resection synchronous colorectal metastasis	3
Ileo-anal pouch	2
Colostomy reversal	1
Number of unplanned operations	196 (84 %)
Anatomical location	
Upper GI	5 (2 %)
Lower GI	98 (45 %)
HPB	35 (16 %)
Abdominal wall	49 (23 %)
Vascular	3 (1 %)
Other	27 (13 %)
Indication repeat surgery	
Malignant disease recurrence	49
Incisional/parastomal hernia	41
Emergency laparotomy	19
Adhesion-related surgery	11
Ostomy closure	
Protective loop ileostomy^a^	35
Colostomy	1
Ileo-anal pouch	3
Relocation ostomy	14
Ostomy creation	2
New malignancy	5
Other	58
Department performing the operation	
Surgery	225
Gynecology	5
Urology	4

^a^Three patients required the formation of a protective loop ileostomy during a reoperation and subsequent closure

**Table 3:** The incidence of unplanned repeat abdominal surgery stratified for anatomical location of index operation

Unplanned repeat abdominal surgery	Anatomical location of index operation				
	Upper GI	Lower GI	HPB	Abdominal wall	Other
No	59 (84 %)	200 (76 %)	82 (79 %)	77 (76 %)	52 (81 %)
Yes	11 (16 %)	64 (24 %)	22 (21 %)	25 (25 %)	12 (19 %)

**Table 4:** Univariable logistic regression analysis for unplanned repeat surgery

Patient factor	OR	95 % CI OR		*p*
Sex				
Male	Ref.			
Female	1.60	1.09	2.35	0.02
Age	0.98	0.97	0.99	<0.01
BMI	0.97	0.92	1.01	0.13
Smoking status				
Non-smoker	Ref			
Ex-smoker	1.12	0.73	1.72	0.59
Smoker	0.87	0.49	1.54	0.64
Physiologic-POSSUM score	0.94	0.90	0.98	0.01
ASA score				
1	Ref			
2	0.92	0.56	1.53	0.75
3	0.58	0.30	1.10	0.10
Diagnosis of IBD	1.17	0.64	2.12	0.62
Number of previous laparotomies				
0	Ref			
1 or 2	1.39	0.88	2.17	0.16
≥3	1.61	0.96	2.71	0.07
Previous laparoscopy				
Yes	1.17	0.66	2.07	0.59
No	Ref			
Anatomical location previous surgery				
Upper gastrointestinal tract	0.83	0.34	2.08	0.70
Lower gastrointestinal tract	1.44	0.98	2.12	0.07
Abdominal wall	1.23	0.77	1.98	0.39
HPB	0.76	0.39	1.46	0.41
Other	1.07	0.72	1.61	0.61
Malignancy as indication for surgery				
Colorectal	0.71	0.41	1.21	0.21
Hepatic	1.67	0.96	2.91	0.07
Esophageal	0.16	0.04	0.68	0.01
Other	0.79	0.44	1.41	0.42
Benign indication for surgery				
Ventral hernia	1.06	0.64	1.76	0.81
Fistula	1.66	0.79	3.49	0.18
Other	1.29	0.86	1.93	0.21
Anatomical location index operation				
Other	Ref			
Lower GI	1.37	0.89	2.11	0.15
Abdominal wall	1.39	0.80	2.43	0.24
Surgical approach				
Median	Ref			
Subcostal	0.94	0.54	1.64	0.82
Other	1.15	0.64	2.06	0.82
Laparoscopy	0.40	0.17	0.96	0.04
Adhesiolysis time				
0–30	Ref			
≥31	1.47	0.94	2.29	0.09
Zühlke score underneath incision				
No or mild adhesions	Ref			
Severe adhesions	1.54	1.02	2.31	0.04
Zühlke score operative area				
No or mild adhesions	Ref			
Severe adhesions	1.53	1.02	2.30	0.04
Zühlke score other abdominal areas				
No or mild adhesions	Ref			
Severe adhesions	1.62	1.04	2.53	0.03
Iatrogenic injury due to adhesiolysis				
Enterotomy	2.19	1.07	4.47	0.03
Seromuscular injury	1.25	0.77	2.02	0.36
Other organ injury	1.18	0.56	2.49	0.66
Wound classification				
Clean	Ref			
Clean-contaminated	1.01	0.68	1.52	0.95
Contaminated	1.07	0.48	2.39	0.87
Dirty	1.78	0.16	20.00	0.64
Ostomy	1.41	0.87	2.27	0.16
Duration of surgery	1.00	1.00	1.00	0.41
Intraabdominal complication	1.65	0.96	2.84	0.07
Relaparotomy	1.52	0.81	2.87	0.20

**Table 5:** Multivariable logistic regression analysis with backward selection for unplanned repeat surgery; Nagelkerke *R* ^2^ = 0.091

Patient factor	OR	95 % CI OR		*p*
Sex				
Male	Ref			
Female	1.53	1.01	3.18	0.046
Age	0.98	0.96	0.99	<0.01
Hepatic malignancy	2.08	1.00	4.33	0.049
Esophageal malignancy	0.21	0.05	0.89	0.034
Surgical approach				
Median	Ref			
Subcostal	0.56	0.26	1.16	0.119
Other	0.92	0.50	1.70	0.785
Laparoscopy	0.26	0.10	0.69	0.007
Intraabdominal complication	1.75	0.99	3.10	0.053

### Univariable logistic regression for risk factors of requiring unplanned repeat abdominal surgery

Female gender (OR 1.60; *p* 0.02), severe adhesions underneath the incision (OR 1.54; *p* 0.04) at the operative area (OR 1.53; *p* 0.04) and other abdominal areas (OR 1.62; *p* 0.03), and an iatrogenic enterotomy (OR 2.19; *p* 0.03) were significantly associated with an increased risk for undergoing repeat abdominal surgery. Three or more previous laparotomies (OR 1.61; *p* 0.07), lower gastrointestinal tract as the anatomical location of previous abdominal surgery (OR 1.44; *p* 0.07), hepatic malignancy as indication for surgery (OR 1.67; *p* 0.07), more than 30 min of adhesiolysis (OR 1.47; *p* 0.09), and intraabdominal complications (OR 1.65; *p* 0.07) showed a trend toward an increased risk for repeat abdominal surgery. Higher age (OR 0.98; *p* < 0.01), higher Physiologic-POSSUM score (OR 0.94; *p* 0.01), esophageal malignancy (OR 0.16; *p* 0.01), and laparoscopic surgery (OR 0.40; *p* 0.04) were significantly associated with a reduced risk for undergoing repeat abdominal surgery. A trend for reduced risk for repeat abdominal surgery was seen for patients with an ASA score of 3 (OR 0.58; *p* 0.10).

### Multivariable logistic regression for risk factors for undergoing unplanned repeat abdominal surgery

Female sex (OR 1.53; *p* 0.046) and hepatic malignancy as indication for surgery (OR 2.08; *p* 0.049) were significantly associated with an increased risk for undergoing unplanned repeat abdominal surgery. Older age (OR 0.98; *p* 0.002), esophageal malignancy (OR 0.21; *p* 0.034), and laparoscopic surgery (OR 0.26; *p* 0.007) were significantly associated with a reduced risk of undergoing repeat abdominal surgery. The area under the curve, representing the predictive value of the variables incorporated in the multivariable logistic regression analysis, was 0.67 (95 % CI 0.62–0.72).

## Discussion

Our results show that one in four patients will require repeat abdominal surgery within 4 years after elective abdominal surgery. Female sex and hepatic malignancy had an increased risk for unplanned repeat abdominal surgery. Older patients, patients undergoing laparoscopic surgery, and patients with esophageal malignancy as the indication for surgery had a significantly lower risk for unplanned repeat abdominal surgery.

The incidence in our study, with a median of 4-year follow-up, was higher than that of a US population-based study of predominantly colorectal procedures with a follow-up of 2 years (14 %) but lower than a population-based 10-year follow-up study of patients undergoing a first abdominal operation in Scotland in the year 1986 (36.7 %), nicely demonstrating the effect of time on incidence of repeat surgery [6, 7]. In contrast to these studies, we utilized detailed and accurate baseline data of a prospective cohort study with real-time assessment of the index operation, providing us with the opportunity to reliably assess the incidences of and the majority of risk factors for repeat abdominal surgery. Although our cohort is drawn from a tertiary referral center and therefore contains more complex abdominal surgery, the results of the abovementioned studies suggest that patients undergoing surgery in a secondary care hospital have a similar incidence of repeat abdominal surgery.

The attrition bias of this study is low with a 88 % completed follow-up of patients included in the study. Although there are significant differences at baseline, with regard to age, diagnosis of inflammatory bowel disease, and esophageal malignancy, these differences are small which reflects the large sample size rather than meaningful differences that would affect our results. Databases of Dutch general practitioners keep nearly complete medical records of patients, including correspondences from hospital admissions, making the results of our study very reliable.

Most data on risk factors for repeat operations, albeit scarce, are from studies in patients with a ventral hernia or inflammatory bowel disease that only report on repeat surgery for disease recurrence [11, 12, 14, 15]. This only accounts for approximately half of all repeat operations according to our results, meaning that previous studies suffer from underreporting of risk factors for repeat surgery in general. For patients with a ventral hernia, size of the defect, previous repair, and an open approach increased the risk of repeat surgery, whereas older age decreased the risk for recurrent hernia repair [11, 15]. Disease-specific patient factors could improve the predictive value of our analysis; due to the heterogeneity of our population, we did not incorporate all disease-specific factors which is a limitation of our approach to include all types of abdominal surgery in the study. Our study also showed that older age was correlated with a lower incidence of repeat abdominal surgery. Young patients have a higher lifetime risk for developing new disease that may require abdominal surgery and are also more often fit for subsequent surgery, explaining that patients of older age have a reduced risk for requiring repeat surgery. Patients who have had a hepatic resection for a malignancy have about a twofold increased risk for a repeat operation. Most likely, this will be a subsequent liver procedure because more than half of patients develop a recurrence within 2 years of whom 40 % is eligible for a reoperation [16, 17]. Patients who underwent a laparoscopic procedure had a significantly decreased risk for repeat surgery; however, only a small number of patients in this series underwent a laparoscopic procedure, and these patients mostly had an uncomplicated medical history. This result should be interpreted with caution. Female gender was an independent risk factor for unplanned repeat abdominal surgery. This result is undoubtedly attributed to the risk of gynecological operations and probably a higher incidence of gallstone disease and pelvic (floor) disorder in women [18, 19].

Around 15 % of the total amount of repeat abdominal operations were loop ileostomy closures. It is debatable to consider loop ileostomy closures as repeat abdominal surgery, as they are viewed as minor procedures. However, during a loop ileostomy closure, the peritoneum is opened and adhesiolysis might be necessary. Furthermore, a systematic review showed that the overall morbidity of a loop ileostomy closure is 17 % and that 4 % of patients undergoing a loop ileostomy closure require a laparotomy [20]. Therefore, we accounted loop ileostomy closures in our study as repeat abdominal surgery. Most ileostomy closures were staged procedures (91 %) and were not incorporated in the analysis assessing risk factors for unplanned repeat abdominal surgery. In our cohort, 9 % of the patients underwent a laparoscopic procedure; this is somewhat low compared to today’s surgical practice. However, the most common indications for repeat abdominal surgery were malignant disease recurrence or other indications, both are unaffected by the surgical approach of the index surgery. The incidence of a ventral hernia or a small bowel obstruction is lower after a laparoscopic procedure [9, 21]. However, the incidence of a ventral hernia is still 10.8 %, and the incidence of small bowel obstruction is 5.5 % 3 years after laparoscopic surgery. In our study, these two indications comprise a minority of indications for requiring repeat abdominal surgery.

An important key finding in our study is that nine out of ten repeat operations are unplanned and almost half is unrelated to the index operation. These results cause a paradigm shift, implicating that the potential benefit of adhesion barriers is not confined to two-stage procedures and disease with known high risk for reoperations for small bowel obstruction or ventral hernia. The high rate of unplanned reoperations suggests a potential for adhesion barriers to reduce morbidity and health care costs due to adhesiolysis-related complications. The effectiveness of anti-adhesive barriers has been demonstrated in a systematic review and meta-analysis showing reduced operative time and a decreased incidence of adhesions up to 50 % [10, 22]. The in-hospital costs are around US$4500 higher for patients requiring adhesiolysis during surgery compared to patients not requiring adhesiolysis [5]. A study assessing the cost-effectiveness of anti-adhesive barriers showed that barriers costing £50 would pay back the cost of its investment if it reduced adhesion-related readmissions for small bowel obstruction by 16 % [23]. Even greater benefits might be gained from reducing adhesiolysis-related complications during repeat abdominal surgery and could be as high as US$927 after open surgery and US$380 after laparoscopic surgery [5, 9, 24].

Our study showed that most reoperations involve the lower gastrointestinal and hepatic-pancreatic-biliary tract and the abdominal wall. In general, younger patients and female patients might benefit most from anti-adhesive barriers, as they have the highest risk for unplanned reoperations. Patients undergoing a second hepatic resection suffer from increased operative time and a higher incidence of organ injury, mostly due to adhesiolysis [25, 26]. A clinical trial assessing the efficacy of an anti-adhesive barrier in two-stage hepatic resection found a reduction in the extent and severity of adhesions as well as a reduction in time needed to mobilize the liver. A trend was seen toward less postoperative complications after the second hepatic resection [25]. Patients who are operated upon because of a hepatic malignancy might benefit from placement of an anti-adhesive barrier around the liver because they have a twofold increased risk for requiring repeat surgery, consisting mainly of repeat hepatic resections.

## Conclusion

In our cohort, one in four patients will require repeat surgery within 4 years after elective abdominal surgery mostly due to malignant disease recurrence, incisional or parastomal hernia, or reasons unrelated to the index operation. The lower gastrointestinal tract, hepato-pancreatico-biliary tract, and the abdominal wall are anatomical locations predominately involved at repeat abdominal surgery. Lower age, females, and patients with a hepatic malignancy show the greatest risk for requiring repeat abdominal surgery. Results may guide cost-effective use of anti-adhesion barriers.
